# Reducing delivery insurance costs through risk score model for food delivery company

**DOI:** 10.1038/s41598-024-57548-3

**Published:** 2024-05-14

**Authors:** Diogo Silva Panham, Francisco Louzada, Pedro L. Ramos

**Affiliations:** 1 Institute of Mathematical Science and Computing, University of São Paulo, São Carlos, Brazil; 2 Faculty of Mathematics, Pontificia Universidad Católica de Chile, Santiago, Chile

**Keywords:** Delivery insurance, Insurance costs, Machine learning, Risk classification, Energy science and technology, Mathematics and computing

## Abstract

In this paper, we propose a novel pricing model for delivery insurance in a food delivery company in Latin America, with the aim of reducing the high costs associated with the premium paid to the insurer. To achieve this goal, a thorough analysis was conducted to estimate the probability of losses based on delivery routes, transportation modes, and delivery drivers’ profiles. A large amount of data was collected and used as a database, and various statistical models and machine learning techniques were employed to construct a comprehensive risk profile and perform risk classification. Based on the risk classification and the estimated probability associated with it, a new pricing model for delivery insurance was developed using advanced mathematical algorithms and machine learning techniques. This new pricing model took into account the pattern of loss occurrence and high and low-risk behaviors, resulting in a significant reduction of insurance costs for both the contracting company and the insurer. The proposed pricing model also allowed for greater flexibility in insurance contracting, making it more accessible and appealing to delivery drivers. The use of estimated loss probabilities and a risk score for the pricing of delivery insurance proved to be a highly effective and efficient alternative for reducing the high costs associated with insurance, while also improving the profitability and competitiveness of the food delivery company in Latin America.

## Introduction

Given its continental dimensions and large population, Brazil stands as a fertile ground for a substantial vehicular presence within its borders. In recent times, the emergence of the COVID-19 pandemic has further accelerated the utilization of specific transportation modes, notably motorcycles and bicycles, propelled by the widespread adoption of delivery apps as indispensable tools for work. Consequently, these alternative delivery modalities have played a pivotal role in facilitating both formal and informal employment opportunities. A research study conducted by the Institute of Applied Economic Research (IPEA), utilizing data from the Brazilian Institute of Geography and Statistics (IBGE), sheds light on the pronounced growth in the number of workers embracing delivery apps. According to PCdoB65^1^, in the last 5 years (data collected from 2015), shows that there was a growth of 979.8% Brazilians working for apps that make some kind of delivery.

Consequently, in light of the aforementioned expansion scenario, numerous food delivery enterprises experienced a remarkable escalation in demand subsequent to the COVID-19 outbreak. This surge in demand was quantified at a $24\%$ increase relative to the onset of the pandemic, as discussed by Kercher^2^. During a period characterized by restrictions on physical contact, the role of food delivery drivers has become crucial in ensuring the provision of essential services. With a significant expansion in the number of service providers in this field, companies operating in the food delivery sector have recognized the necessity of enhancing the coverage provided to their drivers. This strategic decision is driven by the inevitability of accidents occurring during the operational process. It is important to emphasize that the food delivery company employs various methods of delivery, but detailed exploratory analysis has revealed that motorcycles are the predominant mode for deliveries. This significant finding steers the focus of our study towards motorcycles, acknowledging their efficiency and dominance in the company’s delivery operations. By proactively addressing these risks, companies aim to prioritize the safety and well-being of their drivers while ensuring the continuity of their delivery services. Furthermore, determining the adequate insurance pricing for food delivery drivers is of paramount importance, as it enables companies to strike a balance between providing adequate coverage and managing costs effectively.

Machine learning methods have played a crucial role in the advancement of insurance pricing. Leveraging their ability to process vast amounts of data and identify intricate patterns, these techniques have brought about a revolutionary transformation within the industry. They facilitate a more precise analysis of risk factors, leading to fairer pricing strategies. Moreover, they contribute to fraud detection, thereby bolstering security for insurers and policyholders alike. Denuit et al.^3^ developed the autocalibration procedure to correct the bias in insurance pricing models that are trained by minimizing deviance. The approach is used to predict claims in insurance using neural networks and generalized linear models. Campos and Antonio^4^ developed a data-driven insurance pricing model for hierarchically structured risk factors. They compare the predictive performance of three models and find that the Tweedie distribution is well suited to model and predict the loss cost on a contract. The profound impact of these methods is undeniable, as they have significantly improved the accuracy of calculations and the overall management of risks (see for instance,^4–6^).

Auto insurance for motorcycles is considered by insurers to be high risk, and is seen by many as an excluded risk. According to Filho^7^, excluded risk is: “a risk is said to be excluded from coverage and for which the insured does not receive any consideration, nor does the insured have, in the event of loss resulting therefrom, any expectation of secondary indemnity”. Therefore, due to the high risk, the price charged and the lack of coverage make the cost of this operation expensive, both for insurers and for companies that contract this service.

This study explores strategies to reduce insurance costs while maintaining adequate coverage for food delivery drivers. Employing a series of methodological approaches, we first applied the Nearest Neighbor Rule Undersampling technique to balance the route database. This ensured equitable representation of different route types in our analysis, a methodology corroborated by Smith et al.^8^. We then used a decision tree to create a claims profile, selected for its ease of interpretation and accessibility to business stakeholders. This approach facilitated the clear identification of primary claims determinants. Next, logistic regression was used to calculate claim occurrence probabilities based on these profiles, chosen for its straightforward implementation and high interpretability. These techniques synergistically created a transparent and understandable risk score model, instrumental in effective negotiation processes. By presenting a novel risk score model, this study significantly contributes to optimizing insurance costs for food delivery companies. Our research offers valuable recommendations and findings, enhancing the understanding and management of insurance premiums in the context of food delivery services.

This case study examines a rapidly expanding food delivery company, which has seen a significant increase in its delivery driver base due to recent growth. To safeguard these drivers, the company partnered with an insurance provider to extend coverage across all operational routes. However, the rising demand led to increased insurance costs, prompting a detailed investigation to reduce these expenses while ensuring adequate coverage. By ’premium’, we refer to the amount charged by the insurance company for risk coverage and related costs, as outlined by Canôas^9^. The primary objective of this study is to identify strategies that balance cost reduction with comprehensive insurance coverage for the drivers. To address this challenge a combination of machine learning techniques and statistical models was employed to construct a risk score. These methods were selected for their technical robustness and interpretability, essential for making the results accessible to a broad audience.

The remainder of this paper is presented as follows: Section “Literature review” presents a literature review, providing some basic concepts of insurance premium composition and Machine Learning and sampling techniques. In Sects. “Methodology” and “Modeling”, the development of the study will be presented, with the application of the techniques mentioned earlier. In Sect. “Results”, the pricing calculation is performed based on the results of the techniques applied in Sect. “Modeling” and finally, we have the conclusions concerning the study and the results presented in Sects. “Modeling” and “Results”.

## Literature review

The use of statistical models and machine learning techniques plays a key role in in insurance pricing. Jørgensen and Souza^10^ and Smyth and Jørgensen^11^ demonstrated that the Tweedie distribution couble be used model insurance data with significant heterogeneity, overcoming limitations of traditional models such as Poisson and Negative Binomial. Ohlsson and Johansson^12^ explore the application of the Tweedie distribution in generalized linear models for non-life insurance pricing. Denuit et al.^3^ explore the use of self-calibration and Tweedie dominance for insurance pricing with machine learning. They demonstrate that this approach can be effective in dealing with the heterogeneity of insurance data, producing more accurate and robust models. On the other hand, insurance data with hierarchical structures or multifaceted risk factors have proven to require more sophisticated approaches. For instance, Campo and Antonio^4^ propose a unified method for modeling risk factors in workers’ compensation insurance, combining statistical and machine learning models with random effects to handle the complexity of these data. Stassen et al.^13^ also contribute in this area, presenting a unified approach for modeling classification factors in workers’ compensation insurance, demonstrating the effectiveness of this method for handling hierarchical data.

Henckaerts et al.^14^ and Henckaerts et al.^15^ provided promising examples of practical applications of tree-based models for insurance pricing. These studies demonstrate the efficacy of these models in real-world scenarios, achieving high accuracy and interpretability. Yang et al.^16^ also explore the potential of Gradient Tree-boosted Tweedie Compound Poisson models for predicting insurance premiums. The interpretability of models is crucial for understanding by decision-makers and acceptance by regulators. Frees and Valdez^17^ highlight the importance of interpretability in hierarchical models for insurance, while Blier-Wong et al.^18^ offers a comprehensive review of the use of machine learning in P &C insurance, emphasizing the need for transparent and understandable models.

Despite the advancements, the application of machine learning in insurance pricing faces challenges. Dastile et al.^19^ warn about the importance of correcting data imbalance in scenarios with unbalanced classes, avoiding biases that may compromise the fairness and equity of pricing. Wüthrich^20^ discusses bias regularization techniques in neural networks for general insurance pricing. Delong et al.^21^ propose improvements in accessibility for the Tweedie’s Compound Poisson model, making it more user-friendly for practical use. Denuit et al.^22^ provide a solid foundation for claims count modeling, addressing topics such as risk classification, credibility, and bonus-malus systems, relevant to the development of robust machine learning models.

The highlighted research focuses on developing machine learning models and advanced statistical methods for insurance pricing, aiming to increase precision and minimize bias in the data. These studies are primarily centered on the needs of insurers. However, the techniques developed also aim to serve the insurance contracting party, providing tools for better negotiation and substantiation in cases of insurance with excluded risks. This innovative focus, coupled with techniques easily interpretable by business areas, positions this research as a significant milestone in the field of risk modeling.

In this research, the Nearest Neighbor Rule undersampling technique was implemented to correct the data set imbalance, a critical step to ensure the neutrality of the machine learning model in relation to class predominance. This step is crucial to prevent the model from improperly favoring the majority class, which could affect the validity of the results in broader scenarios. Moving away from traditional methods, Decision Trees were adapted to form clusters, a methodological choice noted for its ability to enhance the precision of predictive models. The intuitive graphical representation of these trees aids in understanding decision criteria, essential for effective communication and clear operational understanding among professionals.

Continuing the analysis, the identified clusters were used as independent variables in a Logistic Regression model, devised to estimate the probability of claims occurrence. The determination of risk scores based on this model provides a quantitative foundation for insurance pricing, aligning the company’s pricing strategies with the assessed risks. This process is an integral part of risk management, allowing financial decisions to be based on robust data and precise analysis.

## Methodology

Here, we will discuss key points of our research approach, covering a range of critical aspects. This section is designed to provide a clear overview of the methods and analytical techniques employed, ensuring a transparent and rigorous foundation for our study. We aim to present a concise yet comprehensive explanation of our methodology, allowing readers to grasp the depth and scope of our research process.

### Route analysis

Routes are understood as the path taken by the delivery person. Upon conducting initial analysis, it is evident that motorcycle usage accounts for approximately 83% of the routes, followed by bicycle deliveries at 13%. Another relevant finding observed during the analysis was the data imbalance when separating it into insurance claims and non-claims. The total number of claim records was 396, while the non-claim records reached millions. Additionally, it is worth noting that the majority of routes occur in the afternoon and evening.

### Analysis of delivery person information

The age range falls between 18 and 30 years. However, it is important to highlight that the current database presents inconsistencies, as 38% of the information regarding the age of the delivery persons is missing. There are entries in the database with delivery persons above the age of 80, raising doubts about the reliability of this field in the table. Currently, the process of storing delivery person information is done through Optical Character Recognition (OCR) technology, which extracts information from the driver’s license through a photo. However, the quality of the image can impact the reading process, resulting in records with errors due to image reading failures. Due to the limited information available about the delivery person in the database, which is restricted to the information present only in the driver’s license, and in compliance with the LGPD (General Data Protection Law), which ensures the confidentiality of personal information, it was decided to focus solely on the statistical information related to the routes.

### Hypothesis testing

The statistical procedure known as hypothesis testing involves analyzing an assumption made about a population parameter. Its objective is to evaluate the probability of this hypothesis being true, using data from a sample. This sample can come from a larger population or a data-generating process. For testing qualitative variables, the Chi-Square test was used. The Chi-Square Test for Independence examines the relationship between categorical variables, assessing if they are interdependent or associated. This non-parametric method employs a contingency matrix for data examination. Within this matrix, data is segmented based on two distinct categorical variables: one’s categories are displayed row-wise, and the other’s column-wise. Both variables should encompass two or more categories. Each matrix cell denotes the cumulative number of instances for a designated category combination. The p-value associated with each term probes the hypothesis that the coefficient remains neutral (void of impact). If the p-value is below 0.05, it suggests dismissing the base hypothesis. Consequently, a term having a diminutive p-value may hold importance for the model, given its fluctuations influence the outcome variable. Conversely, an elevated p-value indicates a lack of direct correlation between the variable changes and the outcome alterations.

*Significance level: 0.05* -> *95%*

- Region_: 0.0026
- Modal: 0.0002
- Worker_type: 0.0042
- shift: 0.0016
- segmentation: 0.0384
- route_state: ’1.2765e−20
- route_model: 0.0270
- route_type: 0.1526

Logistic regression was adopted for the quantitative variables. Regression analysis is used to establish an equation that describes the statistical relationship between one or more variables and the response variable. In the adopted model, the binary outcome was considered, which corresponds to the values ”claim” (1) or ”no claim” (0). The fields used were:

- time_minute: 0.0001
- average_time: 0.0002
- promotion: 0.9241
- freight_multiplier: 0.6940
- distance_origin_to_destination:0.7331
- distance_to_origin: 0.9630
- fee: 0.0063
- total_distance_to_destination: 0.0059
- total_distance_to_origin: 0.0438
- total_routevduration:0.2461
- max_rank:0.0035
- max_route_lag:0.0076

After the analysis of qualitative variables, those that showed statistical significance were identified: time_minute, fee, average_time, total_distance_origin_to_destination, total_distance_to_origin, max_rank, max_route_lag, region, modal, worker_type, shift, segmentation, route_state e route_model.

### Outlier analysis

The route database is characterized by a large dispersion, which can negatively affect different stages of the process, such as clustering, sampling, and modeling. To minimize the impact of the high data variability and reduce possible errors, an analysis of outlier values was performed, using the z-score formula

1 $$\begin{aligned} z = \frac{x - \mu }{\sigma }. \end{aligned}$$

To apply the z-score formula (Wang et al.^23^), it was necessary to center the data with a mean of zero and set the standard deviation to 1. In this way, any value above or below this threshold is considered an outlier and can be identified. After identifying these outlier values, they were removed from the database to ensure that subsequent analyses would be less influenced by discrepant values.

### Sampling

During the exploratory analysis process, it was possible to identify an imbalance in the data, as the claims database had a reduced number of records compared to the routes database without claims occurrences, which was quite extensive. Therefore, situations may occur where studying the entire population in analysis (i.e., the database of routes without claims) becomes unfeasible or undesirable, making it necessary to extract a representative subset of the population, known as a sample. Sampling is crucial to ensure the representativeness of the results obtained, which, through appropriate statistical procedures, can be used to infer, generalize or draw conclusions about the population in question. Due to limited computational resources and the evident imbalance in the data, which implies variability in the observed events, an Undersampling technique was used.

Undersampling is a method used to deal with class imbalance in which the majority class is reduced to lessen the disparity between categories. Due to limitations in Spark’s Mlib library, a tool commonly used in the company, it was necessary to use a concept similar to the Nearest Neighbor Rule Undersampling algorithm (see, Chawla et al.^24^, for a detailed discussion). The Nearest Neighbor Rule Undersampling (NNRU) algorithm is an undersampling technique that aims to balance imbalanced classes in a dataset. The algorithm is based on the concept that similar examples tend to belong to the same class and the process involves identifying examples from the majority class that have one or more close neighbors in the minority class, i.e., examples from the minority class that are close to examples from the majority class. Then, the examples from the majority class are removed until the number of examples in both classes is balanced.

Based on the NNRU algorithm’s premise, the unsupervised K-means method was used. This algorithm sets random centroids and calculates the distance of data points in relation to these centroids. Points are grouped into clusters based on the shortest distances to the centroids. As the groups formed by clustering have similarities, reducing information in these groups results in minimal information loss, as a group summarizes the necessary information it can add to the study.

After clustering, another sampling technique called ”stratified sampling” was used (see, Lohr^25^, for a detailed discussion). In this type of sampling, the heterogeneous population is divided into homogeneous subpopulations or strata (like the clusters formed in the clustering using k-means). In each stratum, a sample is drawn. The number of strata is initially defined, and the size of each one is obtained. Next, the number of elements to be drawn from the subpopulation of each stratum is specified, which can be uniform or proportional allocation.

### Clustering and data balancing

In the available data set, there are about 27 million routes without claims and only 396 routes with claims, indicating a problematic class imbalance. This situation is common in classification problems, in which the classes are not equally represented. Although most classification data sets have a small difference in the number of instances in each class, in some cases, imbalance is expected.

To solve this imbalance problem, a clustering technique was employed. Cluster analysis, or clustering, aims to group objects so that the objects in the same group are more similar to each other than in other groups (clusters). Here, the K-means clustering technique was used to handle the unbalanced data problem identified in the database. K-means is a widely used clustering algorithm that aims to group a set of objects into k clusters, so that objects within each cluster are similar to each other and different from objects in other clusters (see, Jain^26^, for a detailed discussion).

**Figure 1:**
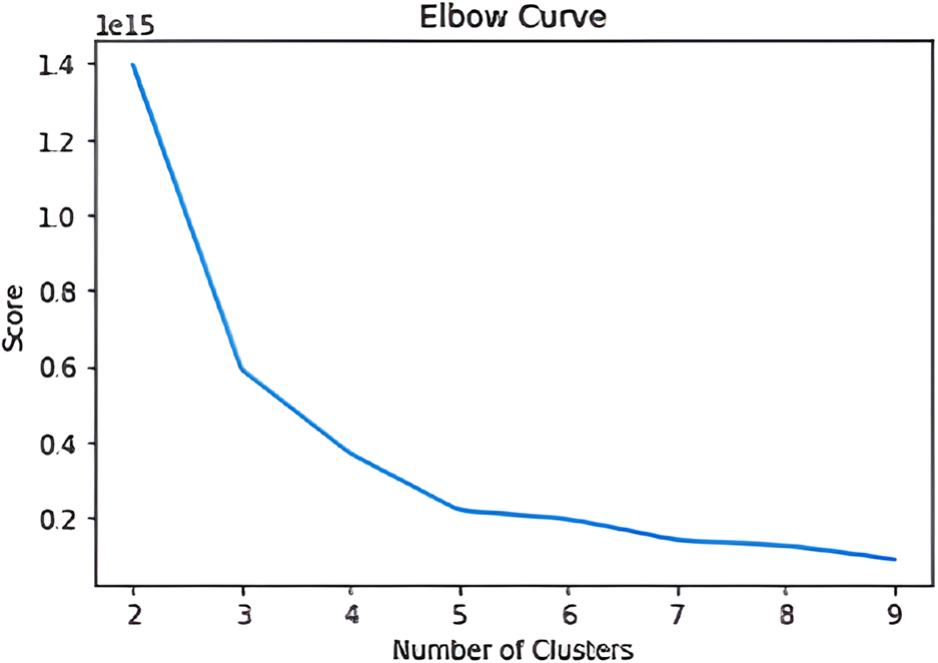
Graph illustrating the optimal number of clusters using the elbow method.

Determining the ideal number of clusters is a critical step in applying K-means. For this, the elbow method was used, which consists of plotting a graph (Fig. 1) of the sum of the squared distances between each point and its closest centroid against the number of clusters. The number of clusters is selected at the inflection point of the curve, where the addition of more clusters does not result in a significant reduction in the sum of squared distances.

The elbow method is a common technique for determining the optimal number of clusters in a data set during the application of the K-means clustering algorithm. Specifically, this method involves running the K-means algorithm for different values of k, while measuring the sum of squared errors for each value of k. It is important to note that to ensure the effectiveness of the algorithm, only quantitative variables were considered in a data set containing more than 27 million routes without claims.

Based on these results, 5 clusters were defined for the analysis (Table 1). This approach provided a clearer and more concise view of the patterns present in the data, allowing for more precise and informed decisions to be made.

**Table 1:** Result of the clustering.

Cluster	Count
2	8118906
0	8609076
1	7647725
4	1132151
3	4247284

The result of K-means was used to generate a stratified sample of the database. Stratification is a statistical technique used to divide a heterogeneous population into subpopulations or homogeneous strata. In this case, stratification was performed using the K-means method. From the stratification, a sample is drawn from each stratum. The formula used to obtain the stratified sample is:

2 $$\begin{aligned} n_i= \frac{N_i}{N}n \end{aligned}$$

where $n_i$ is the sub-stratum, $N_i$ the stratum and *N* the total population.

For the selection of samples in the substratum, a simple random sampling method was used with the aim of obtaining a quantity of records close to the number of records contained in the claims database provided by the insurer. When machine learning and AI algorithms are used in classification methods, it is common to seek a balanced training base, with the same proportion of samples for each class. In databases with dichotomous classes, this proportion would be 50% for each class. To achieve this balanced training base, it is necessary to adjust the sample size of the majority class (routes without incidents) to the size of the minority class (routes with incidents). From the application of the previously mentioned formula, it was possible to reduce the data base and arrive at the ideal sample size for the use of Decision Tree and Logistic Regression algorithms.

## Modeling

Before starting the model with the decision tree, a Multicollinearity test was carried out (Menzel^27^), which consists of a common problem in regressions, in which the independent variables present exact or approximately exact linear relations. For this, a correlation matrix of the quantitative variables was constructed. A significant correlation was observed between the variable ”total route duration” and the variables ”total distance” and ”average_time”, and also between ”time_minutes” and ”average_time”. In the first relationship, it was decided to consider the variable ”total distance”, while in the second relationship, it was decided to consider the variable ” average_time”.

### Model development

The Decision Tree Algorithm was employed to generate claim profiles and validate the consistency of the data. Furthermore, the algorithm offers a method of validation for the variables used, known as ”feature importance.” In developing the risk score model, the decision tree method was selected for its efficacy in segmenting data into meaningful clusters. This approach utilizes the nodes and leaves of the tree to categorize various risk factors, effectively grouping similar risk profiles. The decision tree’s straightforward algorithm allows for easy interpretation and identification of key variables that influence insurance costs. By isolating and analyzing these clusters, the model directly contributes to understanding and mitigating risk factors, ultimately aiming to reduce insurance costs for the food delivery industry. This methodology aligns closely with the objective of providing a practical tool for cost optimization in insurance strategies.

Based on the sample produced by the previously mentioned balancing procedure, the columns (features, variables) to be used in the decision tree model were selected. The construction of the tree nodes is determined by several methods:

- Entropy: Through entropy, the algorithm analyzes the distribution of the data in the predictor variables concerning the variation of the target variable. The higher the entropy, the greater the disorder of the data; the lower it is, the greater the order of this data when analyzed concerning the target variable.
- GINI Index: Like entropy, the GINI index calculation also checks the distribution of the data in the predictor variables concerning the variation of the target variable, but it uses a different method.
- Regression: In regression problems, the goal is to predict a value, not a class. For this, the tree uses the concepts of mean and standard deviation, allowing for the obtaining of a numerical final result.

These concepts are applied both in the construction of the tree and in the function of Feature Importance. This function, as the name suggests, succinctly summarizes which features used in the model are most relevant and effectively summarizes the scenario being modeled, without loss of information. For the model in question, the following results of the feature importance were presented:

**Table 2:** Overview of feature Importance in the developed model.

	Feature	Importance
19	MOTORCYCLE	35.049406
8	Night	24.224920
4	average_time	20.413633
6	Dawn	9.054848
5	total_distance	5.430839
1	max_rank	3.626361
0	total_route_duration	2.199993
13	Inactive	0.000000
18	CAR	0.000000
17	BICYCLE	0.000000
16	without_segmentation	0.000000
15	Super	0.000000
14	Professional	0.000000
10	Casual	0.000000
12	Passive Churn	0.000000
11	Churn	0.000000
9	Afternoon	0.000000
7	Morning	0.000000
3	freight_multiplier	0.000000
2	max_route_lag	0.000000
20	Motorcycle with trailer	0.000000

It is possible to observe in Table 2 that some features, according to the model, did not demonstrate sufficient information gain. With adjusted parameters, such as the decision tree’s depth level, the features “Motorcycle”, “Night”, “Average”, “Dawn”, “total_distance”, “max_rank”, and “total_route_duration” summarized the claims prediction scenario more adherently.

**Figure 2:**
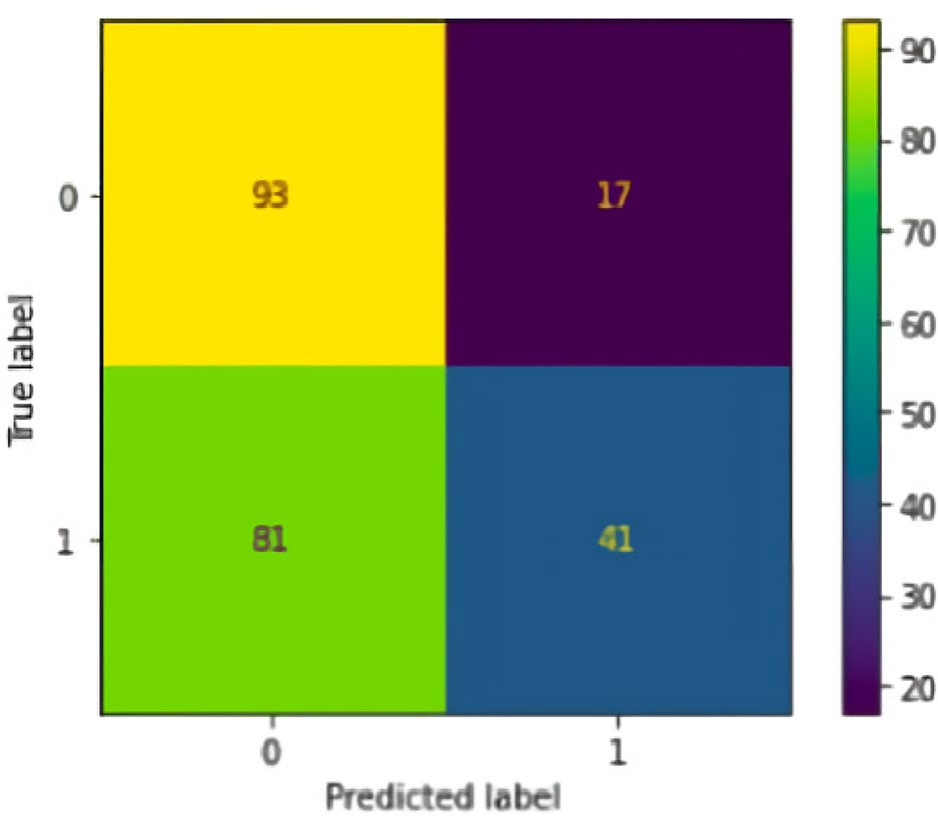
Confusion matrix of the classification model’s performance.

After training the model, a confusion matrix was generated (Fig. 2). It is worth mentioning that a confusion matrix is a tool used in classification models that shows the classification frequencies for each class of the model. Considering the mentioned example, it will provide us with the following frequencies:

- True Positive (TP): occurs when, in the real set, the class we are looking for was predicted correctly.
- False Positive (FP): occurs when, in the real set, the class we are seeking to predict was predicted incorrectly.
- True Negative (TN): occurs when, in the real set, the class we are not looking to predict was predicted correctly.
- False Negative (FN): occurs when, in the real set, the class we are not seeking to predict was predicted incorrectly.

The following metrics were evaluated to validate the adherence of the model:

- Accuracy generally tells us how precise the model is.
- Precision can be interpreted as: of the cases classified as correct, how many were indeed correct?
- Recall indicates how often the classifier finds examples of a class. If an example belongs to that class, recall tells us how often it is correctly classified.
- The F1 score combines recall with precision to provide a single representative number.

**Figure 3:**
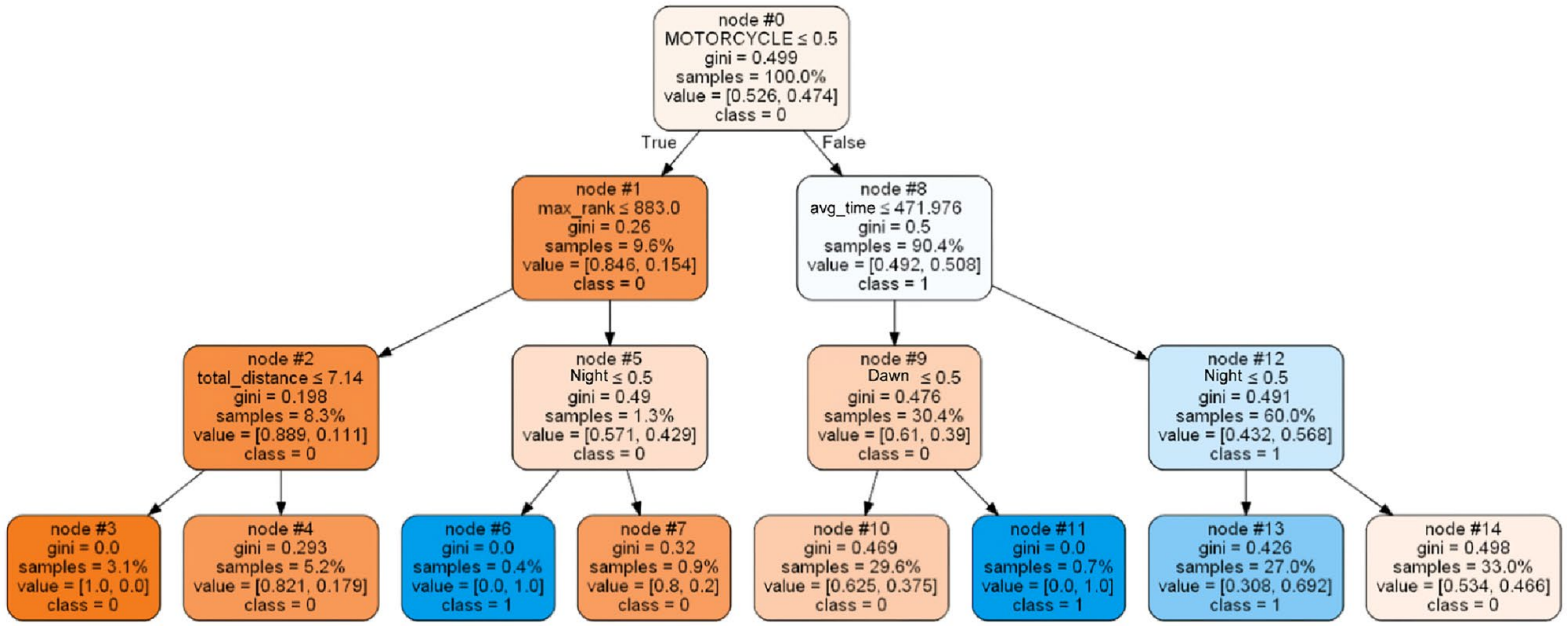
Insurance decision tree for delivery drivers.

The metrics of this Decision Tree related to the confusion matrix are as follows:

From the adjusted results the following decision tree was generated (see Fig. 3):

The decision tree in question (Fig. 3) was generated amid various other scenarios analyzed,being tested by different parameters in the tree (tree depth, criterion: Gini or Entropy, etc.), variables (such as: location, operator, etc). Its validation was carried out together with the business area, and once it presented a logic that reflects the reality of the business, this tree was chosen, despite some model validation metrics that could be improved as presented in Table 3. The careful evaluation of the tree took into consideration its ability to capture the features and behavior of data relevant for decision-making in the context of the business area, specifically financial and operational. This validation with the business area is essential to ensure that the model can provide accurate and useful insights for better steering of strategies and actions in relation to negotiations with the partner insurance company.

**Table 3:** Model evaluation metrics for the decision tree.

	Precision	Recall	F1-score	Support
0	0.53	0.85	0.65	110
1	0.71	0.34	0.46	122
Accuracy			0.58	232
Macro avg	0.62	0.59	0.56	232
Weighted avg	0.63	0.58	0.55	232

### Generalizing the model

Due to the restriction of the size of the claims database, the model was generalized and applied to the database of routes without claims. In this context, the following results were obtained:

**Table 4:** Result of metrics for the generalization of the model.

	Precision	Recall	F1-score	Support
0	1.00	0.91	0.95	480
1	0.00	0.00	0.00	0
Accuracy			0.91	480
Macro avg	0.50	0.46	0.48	480
Weighted avg	1.00	0.91	0.95	480

There was a reduction in the F1-score when generalizing the model, indicating that the evaluation metrics may not be optimal. However, it is important to note that, despite this reduction, the business area validated the model and considered that the presented scenario is consistent with their observations and operational experience. This validation was based on the perception that the decision tree used in the generalization significantly captures the patterns and relevant characteristics of the operational context.

It is worth noting that, although the F1-score was impacted, the accuracy remains at a level of 50%. This measure of accuracy, although modest, indicates that the model still has a reasonable capacity to make correct predictions (Table 4), considering the available data set.

**Figure 4:**
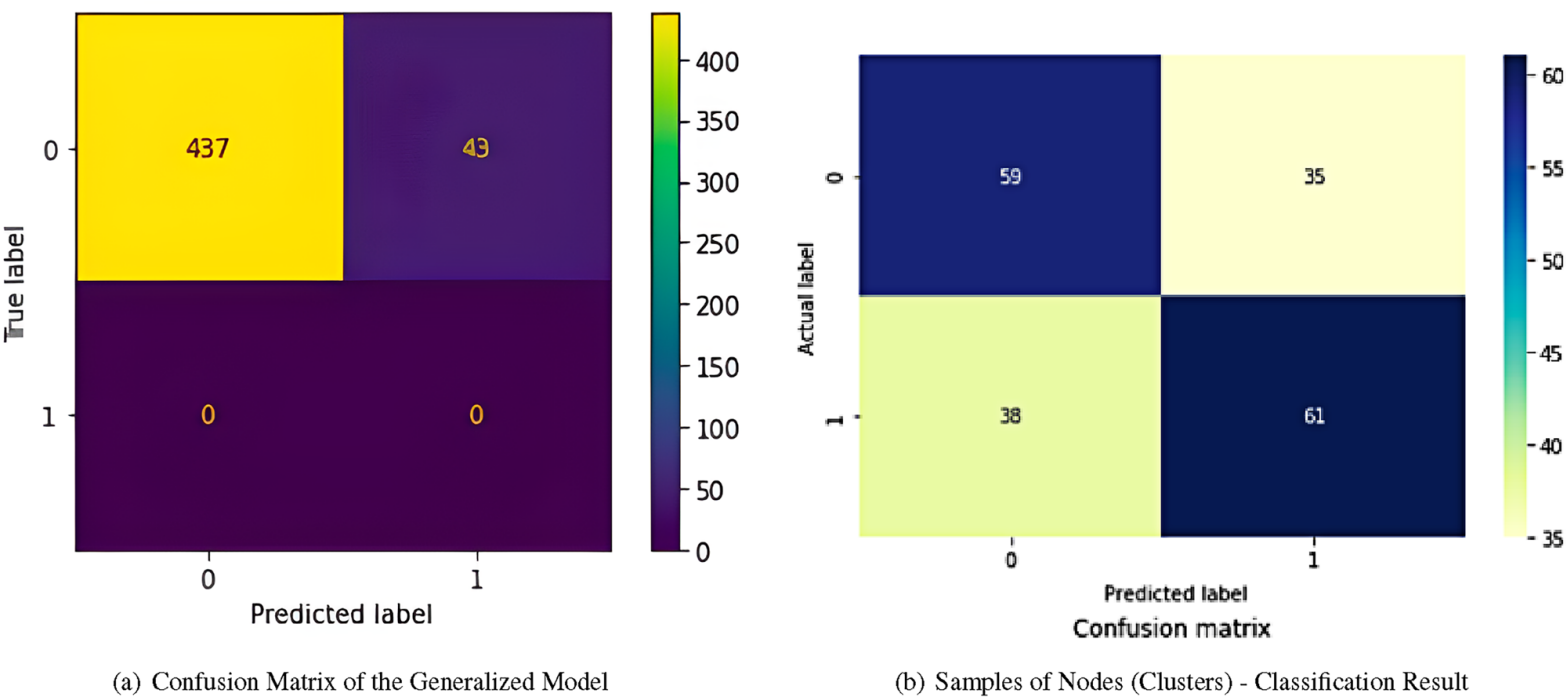
Confusion matrix and classification results.

The confusion matrix is presented in Fig. 4a, which is a tool that allows you to visualize the classification frequencies for each class of the model, providing additional information about the performance of the model in the classification task.

### Logistic regression applied to clusters

After generating the decision tree, the nodes of the tree were used as possible groups to define profiles of deliverers and routes. These nodes were applied to the database and later used in a logistic regression model. For the risk score model, logistic regression was employed to calculate the probabilities of claims based on the defined clusters. This statistical method is adept at handling binary outcomes, making it ideal for predicting the likelihood of claim occurrences. By applying logistic regression to the clusters created from decision trees, we were able to generate nuanced probabilities that reflect varying risk levels. These probabilities were then instrumental in developing a comprehensive risk score, providing a quantifiable measure of risk that directly informs insurance pricing strategies. This approach effectively bridges the gap between data segmentation and practical risk assessment.

The purpose of this procedure was to generate occurrence probabilities for each of the identified clusters, using logistic regression. Through these probabilities, it was possible to obtain a risk score for the routes and deliverers. Logistic regression, in this context, allowed for a more detailed analysis of the characteristics and patterns present in the clusters defined by the decision tree. Based on these analyses, it was possible to assign a risk score to each route and courier, providing a quantitative measure to evaluate the level of risk associated with each one of them.

Thus, the combined use of the decision tree and logistic regression allowed for a more sophisticated approach to defining profiles and assessing risk related to routes and deliverers, providing valuable information for decision-making and strategic planning in the logistics context. As a result, it is possible to observe the confusion matrix in Fig. 4b using a heat map:

The evaluation metrics of the confusion matrix were:

- Accuracy: 0.621761
- Precision: 0.635416
- F1-score: 0.625641

**Figure 5:**
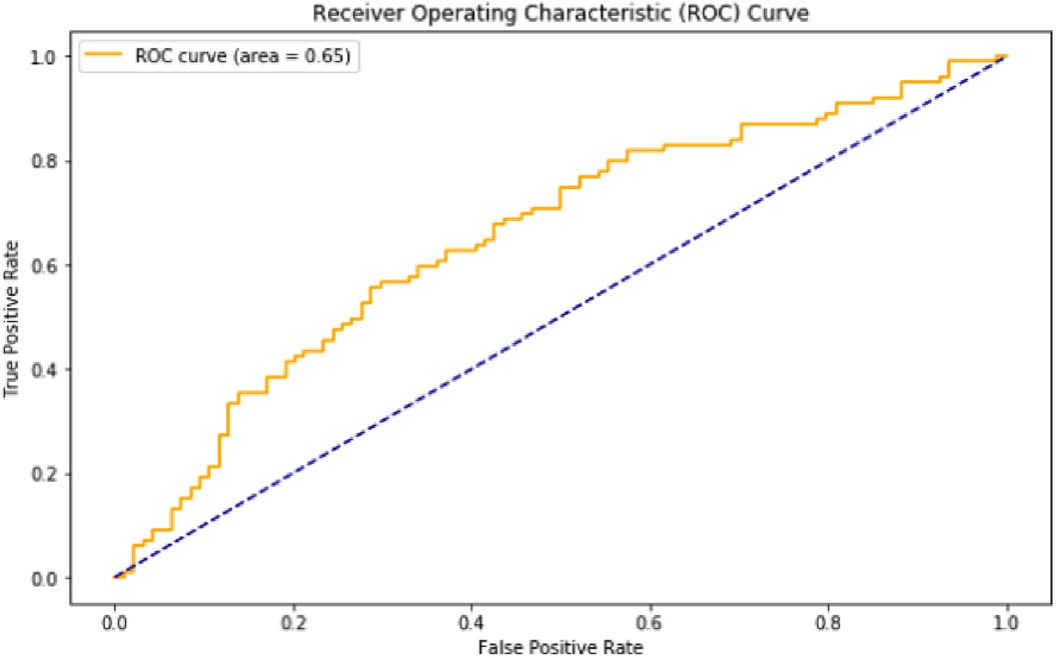
ROC curve of the logistic regression model.

The Receiver Operating Characteristic (ROC) curve is a graphical representation that illustrates the performance of a binary classifier system as its discrimination threshold varies. In the model, the obtained ROC curve can been seen at Fig. 5.

In order to gain a deeper understanding of the model’s results, we proceeded to generate a confidence interval, considering the grouping by node (leaf) of the tree. To evaluate the model, 20 distinct samples were generated, using different seeds. The model was applied to each of these samples, grouping the results by seed and node, in order to analyze the means of the scores for each leaf and calculate the corresponding confidence interval.

The generation of multiple samples with different seeds is a common practice to evaluate the model’s stability and robustness. By conducting this approach, it is possible to observe the variation of the results in different training data sets, which provides a broader view of the model’s performance and allows to identify possible variations caused by the randomness of the selection of training data.

By grouping the results by seed and node, it is possible to calculate the means of the scores for each leaf and subsequently estimate the corresponding confidence interval. This confidence interval provides a statistical measure that indicates the accuracy and reliability of the calculated means, taking into account the variability present in the samples (Table 5).

Thus, the generation of the 20 samples and the calculation of the confidence interval are fundamental steps for the construction of the score and for the statistical analysis of the model’s performance, allowing a more robust and reliable evaluation of its predictive capabilities.

To validate the accuracy and effectiveness of the proposed model, a comprehensive approach was taken, focusing on metrics like accuracy, precision, recall, and F1 score. The prioritization of the F1 score ensured a balance between precision and recall, while overall accuracy was also monitored. This alignment of metrics was crucial for the model’s validation. Additionally, feedback from the business sector was integral, particularly in the construction of clusters. Their day-to-day insights provided a practical perspective, enhancing the model’s relevance and applicability in real-world scenarios. This dual approach, combining statistical rigor with business insights, ensured a robust validation of the model’s performance.

**Table 5:** Samples of nodes (clusters)—classification result.

Seed	Nodes	Average_score	Standard_deviation	Min	Max
8	node 10	0.49	0.11	0.26	0.71
8	node 10	0.58	0.14	0.29	0.86
8	node 3	0.16	0.08	− 0.00102	0.32
27	node 10	0.48	0.12	0.24	0.72
27	node 11	0.64	0.11	0.40	0.86
27	node 3	0.13	0.06	0.0011	0.26
32	node 10	0.48	0.11	0.25	0.70
32	node 11	0.66	0.07	0.51	0.79
32	node 3	0.15	0.08	− 0.012	0.31
59	node 10	0.50	0.12	0.25	0.73
59	node 11	0.63	0.14	0.35	0.91
59	node 3	0.15	0.07	0.009	0.29
93	node 10	0.49	0.11	0.26	0.72
93	node 11	0.57	0.10	0.36	0.76
93	node 3	0.15	0.07	0.004	0.28
404	node 10	0.50	0.11	0.27	0.72
404	node 11	0.71	0.07	0.56	0.85

## Results

Based on the obtained results, two relevant pieces of information emerge: First, each route was classified into nodes, following the structure of the decision tree. Secondly, for each node, a probability of incident occurrence was assigned, ranging from 0 to 1, where 1 represents a $100\%$ chance of an incident. This measure is referred to as a score.

Here, 10 samples were taken, each containing 400,000 routes, with the aim of applying the pricing model. The first analysis carried out on these samples was the calculation of the standard deviation, considering a matrix of nodes by seeds (Table 6). It was concluded that the variations between the nodes and the seeds were minimal, which indicated that the samples could be considered relatively homogeneous.

From this point, one of the 10 selected samples was explored to understand how the nodes could be grouped into more relevant risk classifications for the business context. As a result of this exploration, three risk classifications were identified as can be seen in Table 7: high, medium, and low. These classifications allowed a more tangible approach and more suitable to the company’s needs.

**Table 6:** A summary table of risk definition according to sample analysis.

Node	Average score	Number of routes	Gini coefficient	Participation of total (%)
Node 10	0.44	129734	0.53	32
Node 11	0.61	1829	0.00	0.46
Node 13	0.64	42621	0.57	11
Node 14	0.47	162960	0.50	41
Node 3	0.14	11572	0.00	3
Node 4	0.14	44106	0.71	11
Node 6	0.23	1445	0.00	0
Node 7	0.14	6160	0.68	2
Total	0.35	400427	0.37	100

**Table 7:** Definition of three level risk classification.

Risk level	% of base	Probability of a claim (%)
Low probability	16	20
Medium probability	73	45
high probability	11	63

An important caveat to be highlighted is that, in relation to the business, it is not believed that the proportion of incidents can reach 11% (see, Table 8). However, as indicated by the model, the runs classified as representing 11% of the total have a higher potential for incident occurrence. On the other hand, the runs classified as 3, 4, 6, and 7 have an extremely low risk probability.

Although the value of 11% served as a reference for runs with a high-risk probability, it cannot yet be considered as the final segmentation of our base to be presented to the insurer. It is necessary to assess whether the other samples (see, Table 8) used in the experiment showed a similar behavior regarding critical cases (high risk).

**Table 8:** Risk comparison with the other samples.

Samples	High risk (%)	Low risk (%)	Medium risk (%)
164	11.00	15.80	73.20
223	18.14	16.71	65.15
251	14.89	16.47	68.64
292	15.50	16.69	67.81
410	17.50	17.11	65.39
522	15.73	16.66	67.62
588	15.99	16.73	67.29
590	17.70	16.74	65.55
791	18.66	16.88	64.46
887	17.03	16.58	66.39

Given the results obtained, it was decided to work with the average value of the routes classified as high and low risk. For medium-risk routes, the difference to 100% was calculated to achieve compatibility between values.

This segmentation plays a fundamental role in the study, as it highlights the need to divide the paid values according to the criticality and degree of risk of each route. This will allow a significant reduction in costs, compared to the current scenario, in which the maximum risk - and consequently the highest price - is assumed for all routes.

The model’s design allows decision-makers to understand operational dynamics more effectively, highlighting risk areas and enabling proactive measures to mitigate them. This enhanced understanding is crucial for negotiations with insurance providers, particularly in areas involving excluded risks, where flexibility and cost-effectiveness are limited. The model’s reliance on statistical probabilities for its outcomes offers a solid foundation for these negotiations, potentially leading to more favorable insurance terms and lower costs. This aspect is particularly vital in scenarios where risk-excluded insurance is traditionally expensive and inflexible.

The model effectively classifies routes into various risk levels, assigning each a probability score for incident occurrence. This scoring system, ranging from 0 to 1, was validated through analysis of large data samples, demonstrating consistency across different scenarios. The application of the model revealed three distinct risk categories-high, medium, and low-allowing for more targeted and efficient pricing strategies. This classification not only aligns with the company’s operational needs but also offers a basis for more informed and equitable insurance negotiations. The model’s utility in risk assessment and pricing is demonstrated by its ability to discern and categorize risk with precision, facilitating better decision-making and potentially leading to cost reductions in insurance premiums.

The model’s implementation is intentionally straightforward to ensure usability across various operational levels, including non-technical domains. It provides operational insights and data-driven support for leadership in decision-making processes, particularly in insurance pricing negotiations. This approach aids in cost reduction by equipping leaders with the necessary tools and information to make informed decisions. By demystifying the complexities of insurance pricing and providing actionable insights, the model serves as a valuable asset in optimizing operational costs and enhancing overall risk management strategies.

### Route pricing definition

Once the risk classes and their respective participation in the samples were defined, determining a fair price for each class became necessary to facilitate negotiations with the insurer. The prices, weighted by the number of routes, would then be used to establish a single price.

To gather data for pricing, incident records from January 2020 to February 2021 were examined, yielding the following key findings:

- The average payment per route in recent months was USD 0.08.
- The average cost of claims was calculated as USD 0.05. This value was obtained by dividing the sum of compensated claims and the total open claims in a given month by the total number of routes carried out during the same period. To determine the historical average cost of claims, these monthly values were weighted accordingly.
- It is important to highlight that this study brings a significant novelty to the area of insurance pricing, mainly because it considers the policies already established by insurers. These policies are based on detailed actuarial calculations, which are fundamental for defining the cost of insurance in different situations. By opening up space for discussions on the pricing model, especially in contexts of commercial partnerships, the study offers an opportunity for interested companies to explore and better understand the policies and contracts with their partner insurers. This is vital for them to be able to apply the study effectively and in line with industry guidelines

It is worth noting that this value incorporates both compensated claims and open claims. Consequently, the actual cost is likely lower since only a portion of open claims will be converted into compensation. This suggests that the product may be overpriced, resulting in a USD 0.04 spread (difference between the average cost and the average value of the claim).

Based on these findings, the proposed pricing structure is as follows:

- Low risk: For routes with a low probability of accidents, it is suggested to pay the current cost of the routes plus the open claims, totaling USD 0.05.
- Medium risk: Routes with a medium probability of risk should be priced at an intermediate value of USD 0.06.
- High risk: For routes with a high risk, the payment should remain at the current rate of USD 0.08, which is the pricing currently applied to all routes.

Finally, the final single price was calculated by applying the aforementioned pricing structure to the sample segmentation, resulting in a final value of USD 0.06 per route (see, Table 9). An essential point about pricing that was mentioned is this: a transformation was applied to the results to safeguard the confidentiality of the values and negotiations. This step was taken to ensure the protection of sensitive data during the evaluation process, preventing specific information from being compromised or misused. This demonstrates a commitment to privacy and the integrity of the negotiation process.

**Table 9:** Pricing structure for different risk levels.

Profile risk	%	Price (USD)
Low risk	17	0.05
Medium risk	67	0.06
High risk	16	0.08
Weighted average		0.06

## Conclusions

The study presented herein explored the challenges a global delivery company faced during a period of exponential growth prompted by the Covid-19 pandemic. The data analysis uncovered a significant surge in delivery routes, instigating elevated insurance payments and necessitating the embrace of a new business model.

While existing research provides insights into insurance pricing, our study approaches the topic with a unique and practical perspective by integrating machine learning and statistical methods with the real-world knowledge of industry professionals. This hybrid approach not only meets stringent technical precision and accuracy benchmarks but also guarantees the relevance and applicability of our model.

Throughout the process of data acquisition, analysis, modeling, and consolidation of results, the active participation of the business team played a pivotal role in validating our findings and insights. There remains scope for further enhancements, such as the incorporation of additional variables or fine-tuning of model evaluation metrics, but the model developed aligns neatly with business requirements, as demonstrated by its implementation and score construction executed by the finance team. Significantly, the project’s results were presented to the insurer and effectively facilitated the negotiation of a new pricing contract. The deployment of the proposed model led to substantial cost savings, exceeding millions of dollars relative to previous payments. This outcome underscores the efficacy of the new model and signifies the apex of this study, supplying a robust and efficient solution for the global delivery company.

Overall, this study expands the academic literature by introducing an approach that enhances model accuracy while taking into account the industry’s pragmatic aspects. The fusion of advanced methodologies with empirical knowledge illuminates the value of narrowing the divide between theoretical understanding and practical application. The findings and insights elucidated in this paper serve as a valuable resource for future research and practical endeavors in the realm of insurance pricing and business optimization.

### Supplementary Information

Supplementary Information.

## Data Availability

The data that support the findings of this study are available from a Latin American delivery food company, but restrictions apply to the availability of these data, which were used under license for the current study, and so are not publicly available. Data are, however, available by contacting Diogo Silva Panham at diogopanham@ime.usp.br, upon reasonable request and with the permission of the Latin American delivery food company.
