# Author Correction: Reducing delivery insurance costs through risk score model for food delivery company

**DOI:** 10.1038/s41598-024-62510-4

**Published:** 2024-05-22

**Authors:** Diogo Silva Panham, Francisco Louzada, Pedro L. Ramos

**Affiliations:** 1 Institute of Mathematical Science and Computing, University of São Paulo, São Carlos, Brazil; 2 Faculty of Mathematics, Pontificia Universidad Católica de Chile, Santiago, Chile

Correction to: *Scientific Reports* 10.1038/s41598-024-57548-3, published online 14 May 2024

The Funding section in the original version of this Article was omitted. The Funding section now reads:

Francisco Louzada is supported by FAPESP (grant number 2013/07,375–0).

In addition, Pedro L. Ramos was incorrectly affiliated with ‘Institute of Mathematical Science and Computing, University of São Paulo, São Carlos, Brazil’. The correct affiliation is listed below:

Faculty of Mathematics, Pontificia Universidad Católica de Chile, Santiago, Chile.

The original Article has been corrected.

