# Supplementary material for: Reducing delivery insurance costs through risk score model for food delivery company
Source: Sci Rep. 2024 May 14;14:10994. doi: 10.1038/s41598-024-57548-3 (PMC11094087; doi:10.1038/s41598-024-57548-3)
Supplement: Supplementary file 1 — Supplementary Information. [file 41598_2024_57548_MOESM1_ESM.pdf]

Supplementary Material

Diogo Silva Panham[1,*+], Francisco Louzada[1,*+], and Pedro L. Ramos[2,*+]

[1]Institute of Mathematical Science and Computing, University of São Paulo, São Carlos, Brazil
[2]Faculty of Mathematics, Pontificia Universidad Católica de Chile, Santiago, Chile
[*]pedro.ramos@mat.uc.cl
[+]these authors contributed equally to this work

Appendix

In the following, we describe the variables used in the study.

Variables Analyzed

During the analysis and modeling, variables from datasets related to routes, couriers, and a database of claims submitted by the insurer were used. The definitions of variables used were:
Region: The region where the delivery occurred
Modal: Vehicle used by the courier
Worker_type: Courier's contract with the company (e.g., Independent, Cloud Operator)
Shift: Delivery shift (Morning, Afternoon, Evening, and Overnight)
Segmentation: Driver's segmentation at the time (professional, casual, inactive...)
Route_state: Status of the route (Completed or not)
Route_model: Business model (Logistics or Market Place)
Route_type: Type of delivery route
Time_minute: Courier's online time during the day (Calculated field). Subtracting the maximum and minimum time of the courier on a given day to find the time (in minutes);
Average_time: Average: average online time of the courier. Adding up minutes and dividing by the number of days online (calculated field)
Promotion: Name of the promotion (recorded in the Fleet). Some routes have a promotion, a kind of bonus
Freight_multiplier: Dynamic price multiplier applied to the route;
Distance_origin_to_destination: Distance traveled from the last route of the courier on the day to the delivery destination;
Distance_to_origin: Distance traveled from the last route of the courier on the day back to the restaurant
Fee: The fee that the driver receives depending on the route and if there was a promotion on the date;
Total_distance_to_destination: Sum of the distances traveled on the day to the delivery destination;
Total_route_duration: Sum of the total time of the routes on the day
Max_rank: Calculated field for the maximum value based on the ranking column;
Max_route_lag: Calculated field for the maximum value based on the route delay column
